# Fast stereotactic radiosurgery planning using patient-specific beam angle optimization and automation

**DOI:** 10.1016/j.phro.2022.02.009

**Published:** 2022-02-26

**Authors:** Thomas D. Mann, Kundan S. Thind, Nicolas P. Ploquin

**Affiliations:** a Department of Physics and Astronomy, University of Calgary, Calgary, AB, Canada; b Department of Medical Physics, Tom Baker Cancer Center, Calgary, AB, Canada; c Department of Radiation Oncology, University of Calgary, Calgary, AB, Canada; d Department of Medical Physics, Henry Ford Health Systems, Detroit, MI, USA

**Keywords:** Radiosurgery, Organs at risk, Heuristics, Retrospective studies, Particle accelerators, Automation

## Abstract

**Background and purpose:**

Linac-based stereotactic radiosurgery (SRS) planning for multi-metastatic cases is a complex and intensive process. A manual planning strategy starts with a template-based set of beam angles and applies modifications though a trial and error process. Beam angle optimization uses patient specific geometric heuristics to determine beam angles that provide optimal target coverage and avoid treating through Organs-at-Risk (OARs). This study expands on a collision prediction application developed using an application programming interface, integrating beam angle optimization and collision prediction into a Stereotactic Optimized Automated Radiotherapy (SOAR) planning algorithm.

**Materials and methods:**

Twenty-five patient plans, previously treated with SRS for multi-metastatic intracranial tumors, were selected for a retrospective plan study comparing the manual planning strategy to SOAR. The SOAR algorithm was used to select isocenters, table, collimator, and gantry angles, and target groupings for the optimized plans. Dose-volume metrics for relevant OARs and PTVs were compared using double-sided Wilcoxon signed rank tests (α = 0.05). A subset of five patients were included in an efficiency study comparing manual planning times to SOAR automated times.

**Results:**

OAR dose metrics compared between planning strategies showed no statistical difference for the dataset of twenty-five plans. Differences in maximum PTV dose and the conformity index were improved for SOAR planning and statistically significant. The median SOAR planning time was 9.8 min compared to 55 min for the manual planning strategy.

**Conclusions:**

SOAR planning was comparable in plan quality to a manual planning strategy with the possibility for greatly improving planning efficiency through automation.

## 1 Introduction

Stereotactic radiosurgery (SRS) delivers a high biologically effective dose in a single treatment to intra-cranial lesions. High-definition multi-leaf collimator (MLC) systems allow for MLC-based linear accelerator (Linac) treatments in addition to SRS specific systems like Gamma Knife, CyberKnife, and cone-mounted treatments. MLCs improve treatment efficiency over other SRS modalities because of their ability to treat multiple targets using a single isocenter. Following a non-inferiority study by Yamamoto et al (2014), which demonstrated SRS for 5–10 metastases to be non-inferior to SRS for 2–4 metastases, the delivery of large multi-metastatic plans has become more common as opposed to whole brain radiation [1], [2]. Some phase III trials are currently underway to directly compare WBRT with memantine and/or hippocampal sparing to multi-metastatic SRS [3], [4]. Hippocampal-sparing is also possible with SRS [5].

A Volumetric Modulated Arc Therapy (VMAT) SRS planning strategy was first introduced by Clark et al. [6]. This template-based planning strategy uses equidistant spaced table angles and dose tuning structures to achieve conformity. Recent studies have demonstrated that beam angle optimization can further improve target conformity and reduce Organs at Risk (OAR) dose [7], [8]. Beam angle refers to the set of gantry, collimator, and table angles used in the plan.

Trajectory-focused Beam Angle Optimization (BAO) is currently divided into three categories based on the degrees of freedom: fixed collimator and table [7], [8], [9], dynamic collimator or table [10], [11], [12], [13], [14], [15], and both dynamic collimator and table [16], [17], [18], [19], [20]. BAO for multi-metastatic SRS with fixed collimator and table during delivery focuses on minimizing the “island blocking” problem as described by Kang et al. [7]. This geometric heuristic measure’s multi-target alignment with MLC leaves in the beam’s eye view, which causes gaps in MLC coverage or “islands”.

Our group previously demonstrated a fast and accurate collision prediction application developed using a treatment planning system Application Programming Interface (API) [21]. This application was used to develop a beam angle optimization algorithm, the first to implement automated patient-specific collision prediction directly into the optimization process. Our solution uses a clinically implementable fixed table and collimator approach. MLC projection summing, similar to Wu et al. [8] is applied to find approximate solutions to the Island Blocking Problem. Additional geometric metrics are used, including OAR overlap and gantry range. Current BAO algorithms are limited by lack of collision prediction during the optimization process. Either the treatment space is reduced based on rough estimates of available space, or physical measurements need to be made for every patient. This represents a significant hurdle for clinical implementation.

Stereotactic Optimized Automated Radiotherapy (SOAR) is an efficient alternative to a manual planning strategy. Automation of the optimization process using the treatment planning system API allows direct access to patient data and the ability to automatically create and edit treatment plans. SOAR streamlines the SRS planning process and automates contour generation, beam placement, and other steps up to the first round of VMAT optimization.

This study builds on previous works that have shown the benefit of beam angle optimization compared to generic plan templates [7], [8], [22]. These studies showed dosimetric benefit, but stopped short of producing a clinically viable solution. Our study overcomes typical barriers to implementation of BAO such as collision risks and increased planning times and aims to show that high plan quality can be achieved without sacrificing clinical efficiency.

## 2 Materials and methods

### 2.1 Beam angle optimization algorithm

Beam angle optimization began with the selection of PTVs, OARs to avoid, and number of isocenters to use. PTVs were automatically assigned to an isocenter group based on proximity. The geometric center of each PTV group was used as the isocenter location. Collision prediction was applied to determine the maximum gantry range at each table angle for a given isocenter. The available treatment space was used for beam angle optimization which included calculation of the MLC scores, OAR overlap scores, and gantry range scores. Optimization results were displayed to the user and optimal beam angles were automatically selected for inclusion using a ranking process based on the MLC, OAR overlap, and gantry range scores.

The optimal collimator angle for each table angle was determined using an MLC score ($Z_{\mathrm{MLC}}$). MLCs were fitted to the outside of target contours in the beams eye view for each gantry trajectory. At each control point i along the gantry trajectory the MLC open field area was calculated by subtracting the left and right MLC locations ($l_{\mathrm{ij}},r_{\mathrm{ij}}$) and multiplying by the width ($w_{j}$) of the *j*^th^ MLC. The MLC score was the total summed area at each control point divided by the number of control points n.

$$Z_{\mathrm{MLC}}=\sum_{i}\frac{\sum_{j}\left(l_{\mathrm{ij}}-r_{\mathrm{ij}}\right)w_{j}}{n}$$

**Fig. 1:**
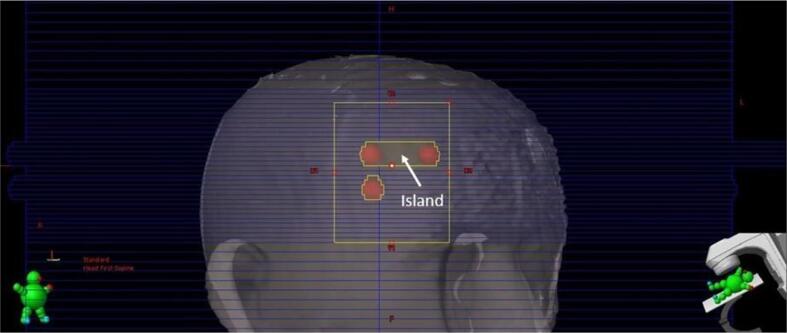
Visual example of the “Island Blocking” Problem. This occurs when multiple PTV structures align with the MLC leaves resulting in a gap of MLC coverage in the center.

This formulation did not correct for the target area contribution to the total open field area. A high MLC score denoted a high degree of “Island blocking” between PTVs while a low MLC score indicated better MLC coverage. An example of the “island blocking” problem can be seen in Fig. 1. Algorithm resolution was limited to 5 degrees for gantry, collimator, and table angles to improve efficiency.

OAR overlap with PTV structures in the beams eye view results in unwanted dose to critical organs. An OAR Overlap score ($Z_{\mathrm{OAR}}$) was used for ranking possible beam combinations [16]. The overlap score measured the percentage of the total PTV area ($A_{\mathrm{PTV}}$) that overlapped with any OAR area ($A_{\mathrm{OAR}}$) for all control points (i).

$$Z_{\mathrm{OAR}}=\frac{\sum_{i}A_{\mathrm{OAR}}\cap A_{\mathrm{PTV}}}{\sum_{i}A_{\mathrm{PTV}}}$$

The maximum possible gantry trajectory for each isocenter and table angle was calculated using a collision prediction algorithm as described in Mann et al. [21]. This algorithm used geometric models of the patient, treatment table, and gantry to predict the maximum trajectory. To account for gantry collisions with the patient body, an anthropomorphic torso model was added to the body contour. The total gantry trajectory was trimmed to exclude gantry angles that treated through selected OARs to reach the target. A gantry range score ($Z_{\mathrm{Gantry}}$) was used to penalize table angles with short gantry trajectories. The gantry range score was determined using a step function based on the total range in degrees.

$$Z_{\mathrm{Gantry}}=f\left(range\right)$$

$$f\left(x\right)=\left\{\begin{array}{c} 0\;\text{if}\;x>350^{{}^{\circ}}\\ 1\;\text{if}\;260^{{}^{\circ}}<x\leq 350^{{}^{\circ}}\\ 2\;\text{if}\;170^{{}^{\circ}}<x\leq 260^{{}^{\circ}}\\ 3\;\text{if}\;80^{{}^{\circ}}<x\leq 170^{{}^{\circ}}\\ 4\;\text{if}\;x\leq 80^{{}^{\circ}} \end{array}\right)$$

After optimization, the algorithm determined the best collimator angle for each table angle based on the MLC score. The algorithm then selected from the set of possible table angles the number required for the treatment plan. The selection process was performed using a ranking system for each beam based on the following criteria: MLC score, OAR overlap score, and gantry range score. The MLC and OAR Overlap score were both sorted from low to high values. The score rank was the index of the score in the sorted list. The lists were the same length so MLC and OAR overlap scores were weighted equally. The gantry range score was intentionally weighted lower. The total score ($Z_{\mathrm{Total}}$) was the sum of the MLC and OAR score rankings and the gantry range score.

$$Z_{\mathrm{Total}}={Rank(Z}_{\mathrm{MLC}})+Rank\left(Z_{\mathrm{OAR}}\right)+Z_{\mathrm{Gantry}}$$

Low scores denoted optimal beams. The algorithm started by selecting the lowest total scoring beam, and iteratively adding lowest scoring beams until the required number was reached. Each beam added was >30 degrees in table angle separation from any beam already selected. This criterion forced the selected beams to be spread over a wider area.

### 2.2 Retrospective plan comparison

Currently our clinic uses a manual planning strategy that starts from a beam angle template. The template is then modified by the treatment planner until the desired dose distribution is achieved. Possible modifications include adjustments to table and gantry angles to avoid OARs, choice of collimator angles, and fine-tuning of VMAT optimization objectives. SOAR optimized plans were compared to the manual planning strategy using a retrospective comparison of previously treated clinical SRS plans. Twenty-four patients were included in the plan comparison with one patient having two treatments included in the study. The number of targets per plan ranged from two to thirteen. Patient plan characteristics are shown in Table 1. Patient data was acquired and used according to institutional ethical standards and regulatory approval. All selected patients were treated within the last two years using a frameless linac-based VMAT stereotactic delivery. Each PTV volume was derived using a 1 mm isotropic expansion of the GTV, which was contoured on T1-weighted gadolinium contrast-enhanced MRI fused to the planning CT. Selected patients were re-planned twice, first, using the manually chosen clinical beam angles re-optimized with the generic template of VMAT optimization objectives in Table 2, and second, using the SOAR algorithm. Keeping VMAT objectives constant allowed us to isolate the impact of the SOAR beam angle selection. SOAR plans used the same number of isocenters and total number of beams as the clinical plans but the isocenter location and PTV groupings varied. Plan sums were used for patients with multi-isocenter plans. Dose was normalized for every plan to ensure that the prescription isodose line (80%) covered 100% of every target volume. Dose metrics for the normal brain tissue, PTV structures, and relevant OARs were extracted for each plan. The Paddick Conformity Index (CI) was used to compare coverage of all PTV targets with the prescription isodose line. Dose fall-off outside the target was assessed using a Gradient Index (GI) defined as the ratio of half the prescription isodose and the prescription isodose equivalent sphere diameters. Statistical comparisons were made between the manual clinical plan and SOAR plan for each metric using double-sided Wilcoxon signed rank tests (α = 0.05, no adjustment for multiple comparisons).

**Table 1:** Patient Plan Characteristics.

Metric	Average	Median	Min	Max
Prescription Dose (Gy)	–	21	18	30*
Number of PTVs	–	4	2	13
PTV Volume [cm^3^]	1.58	0.38	0.05	13.09
Total PTV Volume [cm^3^]	7.06	5.94	0.38	25.57
Off-axis Distance [cm]	3.50	3.67	0	7.66

*max prescription dose was delivered in five fractions.

### 2.3 Planning automation

**Fig. 2:**
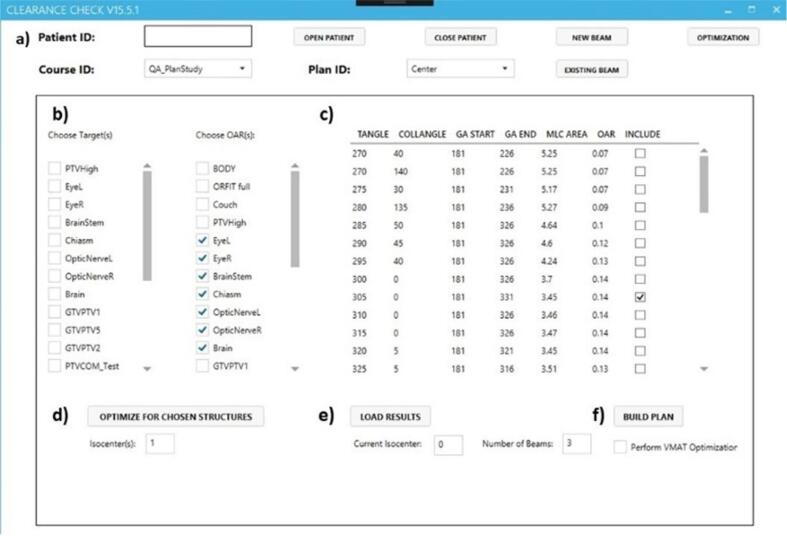
SOAR planning user interface a) Patient, course, and plan selection b) PTV Target and OAR selection c) Optimization results data grid, results for each isocenter are loaded separately d) Isocenter number selection and optimization start button e) Load results button, isocenter selection, and number of beams selection f) Build plan button with option to perform VMAT optimization.

Eclipse Scripting 15.6 allows for the automation of many planning tasks. The SOAR planning user interface can be seen in Fig. 2. After BAO the results for each isocenter were loaded one at a time into the data table. The plan isocenter, gantry trajectories, and beam rankings were determined as described previously. SOAR plans used the same number of beams and optimization objectives as the clinical copy for the retrospective plan comparison. Beam angles were determined using the beam selection algorithm. Changes to the list of selected beams could be made in the optimization results table (Fig. 2. c). Clicking Build Plan (Fig. 2. f) started plan creation using the selected set of beams. Optimization objectives and associated weightings are shown in Table 2. All objectives were automatically added to the plan including a PTV ring structure to limit the 50% prescription dose spread and an automatic Normal Tissue Objective (NTO) to limit low dose spread. The automatic NTO is available in Eclipse 15.5 and uses internal parameters that depend on the distance of high dose areas to the target instead of a user defined accepted dose level curve.

**Table 2:** Generic optimization objectives template used for VMAT photon optimization.

Structure	Objective Type	Volume [%]	Dose [cGy]	Weighting
PTV	Upper	0	1.4 × PD*	150
PTV	Lower	100	PD*	150
GTV	Lower	100	1.1 × PD*	100
Ring	Upper	0	0.5 × PD*	150
OAR	Upper	0	150	50
NTO	Upper	Auto	Auto	150

*PD is prescription dose to the 80% isodose line.

### 2.4 Automated efficiency compared to a manual planning strategy

Planning efficiency was directly compared for a subset of clinical SRS patients. All multi-metastatic SRS cases planned over a period of one month were selected for inclusion. Over the one month accrual period five patients were eligible for this comparison study. The median number of targets was 5 (range: 2 to 13) with 3 patients having two isocenters. Planners using the manual planning strategy were asked to track the time spent on creating the plan, adjusting beam angles, creating dose-tuning structures, and assigning initial optimization objectives. Automated plan creation using the SOAR algorithm was then timed using the same number of isocenters and total number of beams as the clinical plan. Plan quality results from these plans were also included in the retrospective plan comparison.

## 3 Results

**Fig. 3:**
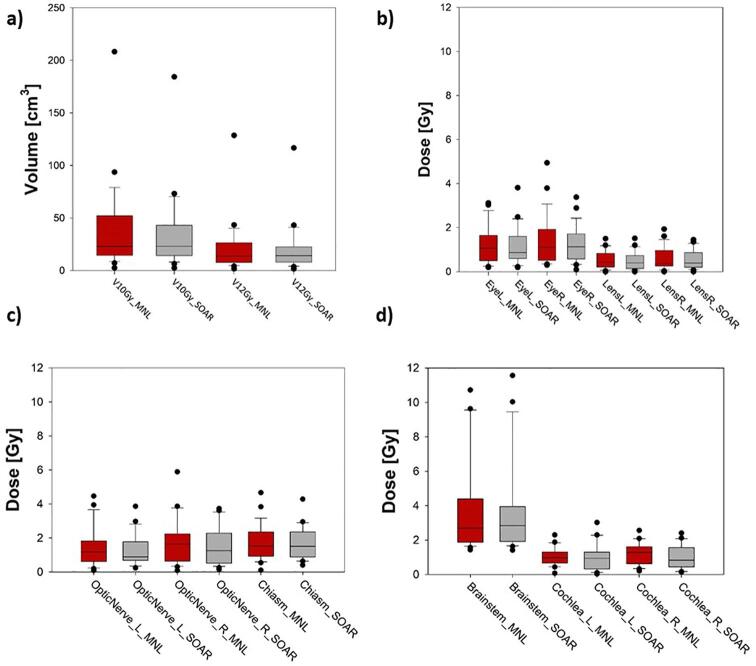
Boxplots of OAR dose metrics comparing manual planning (MNL) in red to SOAR planning (SOAR) in grey. The central line shows the median, edges of the box denote the 25th and 75th percentiles, and whiskers are the min and max points not including outliers. Outliers shown as black circles. a) Normal brain volume receiving 10 and 12 Gy. b) Max doses to the eyes and lenses. c) Max doses to the optic apparatus. d) Max dose to the brainstem and mean dose to the cochlea.

Fig. 3 shows the spread of values for each planning technique and dose metric. A more detailed summary of all dose and plan quality metrics included in the plan comparison study can be found in Table 3. Comparison of twelve different organ at risk dose metrics showed no statistically significant difference between clinical and SOAR plans for all twelve metrics. The healthy brain volume receiving at least 10 Gy (V10Gy) and 12 Gy (V12Gy) had an absolute difference in median volume of 0.1 cm^3^ and 0.4 cm^3^ respectively. Two of the three PTV plan quality metrics were found to be statistically significant favoring the SOAR dataset. Comparing normalized plans, the median PTV max dose (D2%) was reduced for SOAR planning from 138.5% (range: 117.0% to 159.1%) of the prescription dose to 138.1% (113.9 % to 156.4%) (p = 0.003). The median conformity index was 0.71 (0.38 to 0.90) for manual planning and 0.72 (0.39 to 0.90) for SOAR planning (p = 0.04) with a value of 1 being ideal. The median gradient index decreased from 1.85 (1.41 to 3.13) for manual planning to 1.82 (1.39 to 3.45) for SOAR with larger values indicating poorer plan quality.

**Table 3:** Dose metric comparison results for clinical and SOAR plans.

Structure	Dose Metric	N	Manual Plans			SOAR Plans		
			Median	Min	Max	Median	Min	Max
Healthy Brain	V12Gy[cm^3^]	25	13.79	1.62	128.62	14.17	1.57	116.69
Healthy Brain	V10Gy[cm^3^]	25	22.94	2.54	208.26	23.00	2.50	184.30
Eye_L	D0.1cc[Gy]	25	1.06	0.19	3.12	0.87	0.19	3.81
Eye_R	D0.1cc[Gy]	25	1.11	0.30	4.94	1.13	0.09	3.38
Lens_L	D0.1cc[Gy]	24	0.43	0.00	1.50	0.40	0.00	1.51
Lens_R	D0.1cc[Gy]	24	0.38	0.00	1.93	0.39	0.00	1.45
Optic Nerve L	D0.035cc[Gy]	25	1.18	0.08	4.46	0.89	0.23	3.86
Optic Nerve R	D0.035cc[Gy]	25	1.64	0.08	5.89	1.25	0.14	3.73
Cochlea_L	Mean[Gy]	20	0.97	0.07	2.30	0.94	0.03	3.02
Cochlea_R	Mean[Gy]	20	1.28	0.20	2.56	0.83	0.13	2.41
Brainstem	D0.03cc[Gy]	25	2.69	1.43	10.72	2.84	1.41	11.56
Chiasm	D0.035cc[Gy]	25	1.52	0.10	4.66	1.51	0.40	4.28
PTV	D2% [%]	112	138.5	117.0	159.1	138.1*	113.9	156.4
PTV	CI	98	0.71	0.38	0.90	0.72*	0.39	0.90
PTV	GI	74	1.85	1.41	3.13	1.82	1.39	3.45

*Statistically significant results are highlighted in bold.

For the planning efficiency comparison the manual median planning time was 55 min and ranged from 18 min for the two target case to 5 h and 40 min for the thirteen target case. The median SOAR planning time was 9.8 min (range: 5.7 min to 13.2 min). The median time saved was 45.2 min with a total range of 9.4 min to 326.8 min.

## 4 Discussion

This retrospective study compared a manual VMAT planning strategy to an automated solution that uses beam angle optimization, collision prediction, and a treatment planning system API to efficiently create SRS treatment plans for linac-based multi-metastatic radiosurgery. With a cohort of 25 patient plans SOAR planning was comparable in plan quality to manual planning, showing no significant difference in dose metrics for OARs and a statistically significant reduction in PTV max dose and increase in target conformity. A manual planning strategy is highly dependent on the individual planner’s expertise and uses a trial and error process that can be inefficient and inconsistent. Automated processes like SOAR reduce reliance on individual expertise and reduce time required for treatment planning.

Dose metrics relevant to SRS were used to assess plan quality in a retrospective comparison study [23], [24], [25], [26], [27]. All twelve OAR dose metrics showed no statistically significant differences over 25 plans. Comparison of the box plots in Fig. 3 shows the median and range of values for each dose metric to be similar, including most outliers. The difference in median V10Gy and V12Gy dose volumes was less than 0.5 cm^3^ showing similar normal brain tissue sparing. Outliers for V10Gy and V12Gy were reduced by 24.0 cm^3^ and 11.9 cm^3^ using SOAR planning. All dose outliers fell within 1 Gy of each other or had > 1 Gy decrease for SOAR planning. Median values improved for all three PTV metrics with both the PTV max dose and the conformity index being statistically significant. The current study was limited by only having 25 cases and could benefit from a larger patient plan sample.

The real benefit of SOAR lies in the automation of the SRS treatment planning process while still achieving comparable plan quality. Planning time was reduced by 9.4 min to 45.2 min for simpler plans (<6 targets) and up to 327 min for more complex plans (>8 targets) over the five patients sampled. SOAR automated planning took a maximum of 13.2 min compared to 5 h and 40 min for manual planning. Algorithm timing depends on the number of isocenters, size of the targets, and number of targets. The SOAR application is designed as a stand-alone executable, ensuring the planning workspace is available for other tasks while the algorithm is running.

Previous solutions to the SRS island-blocking problem showed a significant reduction in normal brain dose. Kang et al. [7] reported 20% reduction in V12Gy volume for three targets, and 6% reduction for six targets. Wu et al. [8] achieved an approximate 9% average drop in V12Gy volume for three lesions and less than 3% reduction for four or five lesions. It is important to note that this reduction is in comparison to a fixed collimator angle of 45 degrees in both cases, Wu et al. [8] using the same table angles and Kang et al. [7] only using a table angle of zero. Our study used collimator and table angles copied from the original clinical plan for the manual planning cohort. These were chosen by the planner using visual assessment of the target locations in the beams-eye-view, resulting in a closer approximation to the optimal solution than using a fixed angle approach. Similar results were reported in a study by Pudsey et al. [28] which compared optimized to manually selected collimator angles for VMAT-based single-isocenter multiple-target SRS with a retrospective cohort of 10 patients. They found no significant differences between plans with or without collimator optimization.

The SOAR algorithm uses a greedy selection process for the beam angle selection, subject to spreading criteria. Using a probabilistic selection algorithm instead could result in a better solution. Another limitation to our algorithm is the MLC score which does not correct for the PTV area contribution to the total MLC area. Irregular PTV shapes and overlap of PTV regions can also contribute to variations in the total MLC area resulting in an approximate solution to the island blocking problem. For multi-isocenter plans, the SOAR algorithm does not detect overlapping beam angles from separate isocenters. This could potentially lead to an increase in the V12Gy normal brain volume. In an updated version of the application, users can see a 3D visual display of all plan beam angles and manually adjust for overlapping beams.

Past studies have shown various ways to solve the island-blocking problem and compared to a number of different planning methods [7], [8], [22], [28]. This study shows how beam angle optimization can be clinically implemented using a treatment planning system API to increase efficiency while maintaining plan quality. Our study benefits from a larger patient cohort, using 25 patient plans instead of 10, and having a clinically usable application for direct comparison of planning times. The algorithm is implementable in any radiotherapy clinic with access to a treatment planning system API performing MLC-based SRS treatments. In addition, the application can be implemented for any immobilization system, unlike vendor SRS planning solutions which require specific immobilization for collision prevention and limit the number of table angles available for treatment.

This study demonstrated that the SOAR application was able to maintain the high plan quality achieved by manual planners, while improving clinical efficiency through automation. Future work will include a prospective implementation of the SOAR algorithm for multi-metastatic planning at our clinic.

## Declaration of Competing Interest

The authors declare that they have no known competing financial interests or personal relationships that could have appeared to influence the work reported in this paper.
